# A randomised, parallel-group clinical trial comparing bedinvetmab to meloxicam for the management of canine osteoarthritis

**DOI:** 10.3389/fvets.2025.1502218

**Published:** 2025-03-24

**Authors:** John F. Innes, B. Duncan X. Lascelles, Daniel Bell, Robert Tulloch, Alex McVey, Chad Northcott, Mahala Welbourn, Kate Higgins, Veronika Horakova, Thomas W. Maddox

**Affiliations:** ^1^Movement Referrals: Independent Veterinary Specialists, Runcorn, United Kingdom; ^2^Institute of Infection, Veterinary and Ecological Sciences, University of Liverpool, Leahurst Campus, Neston, United Kingdom; ^3^Translational Research in Pain Program, North Carolina State University, Raleigh, NC, United States; ^4^Department of Clinical Sciences, College of Veterinary Medicine, North Carolina State University, Raleigh, NC, United States; ^5^The Sands Veterinary Practice, Apex House, Chester, United Kingdom; ^6^The Market Town Vet, Oswestry, United Kingdom; ^7^Woolton Veterinary Centre, Liverpool, United Kingdom; ^8^Vetcare Ltd., Leigh, United Kingdom; ^9^Wigan Vets for Pets, Wigan, United Kingdom; ^10^The Village Veterinary Surgery, Formby, United Kingdom; ^11^Hollybank Veterinary Centre Ltd., Sandiway, United Kingdom

**Keywords:** bedinvetmab, meloxicam, canine orthopaedic index, canine osteoarthritis, degenerative joint disease, monoclonal antibody, pain management

## Abstract

Bedinvetmab (Librela^®^), a fully canine anti-nerve growth factor monoclonal antibody, was compared to the non-steroidal anti-inflammatory drug (NSAID) meloxicam in dogs for the management of osteoarthritis-related pain in a randomised, open-label, multicentre, parallel-group study. Subjects were recruited from general practices as client-owned dogs with appendicular osteoarthritis. Dogs were block randomised 1:1 to either daily oral meloxicam or bedinvetmab, administered subcutaneously once a month. The primary endpoint for efficacy was the change from baseline in the Canine Orthopaedic Index (COI) score. Linear mixed-effects models were used for statistical analysis conducted on a per-protocol and intent-to-treat basis. We hypothesised that bedinvetmab would demonstrate superior efficacy and safety compared to meloxicam; the number needed to harm (NNH) for meloxicam, relative to bedinvetmab, was calculated. Of the 190 screened dogs, 101 were randomised (bedinvetmab 52; meloxicam 49). Overall, both treatment groups showed a significant reduction in COI scores relative to baseline (*p* < 0.001). The bedinvetmab group experienced a larger mean reduction in COI scores, but this was not statistically significant. A significant effect of the visit was observed, with later visits showing a significantly greater reduction in COI compared to Visit 2 (*p* < 0.001). The bedinvetmab group reported four (AEs), whilst the meloxicam group reported 17, with nine of those being gastrointestinal system disorders. Additionally, more dogs in the bedinvetmab group completed the study (*n* = 44) compared to those in the meloxicam group (*n* = 33). This is the first study to compare bedinvetmab to an NSAID for the management of osteoarthritis-related pain in dogs. The results suggest that both products are equally effective in managing OA pain, with efficacy improving over time for both treatments. Bedinvetmab was associated with fewer AEs. These data will aid clinicians and pet owners in choosing analgesic options for dogs with osteoarthritis.

## Introduction

Osteoarthritis (OA) is the most common orthopaedic disorder in dogs. Prevalence estimates vary depending on the case definition; one retrospective study of practice management system data from multiple primary care practices in the United Kingdom reported an annual period prevalence of 2.5% (1). However, a prospective survey of dogs aged 8 months to 4 years, using radiographic and clinical case definitions, found that 39.8% developed radiographic OA, and 23.6% developed clinical OA (2). Furthermore, in that study, only 2 of 29 dogs identified with clinical OA were receiving pain management. In another study, when dog owners were surveyed with an OA checklist and responded positively to at least one item (*n* = 550), 38% of their dogs were confirmed to have OA after a veterinary examination (3). Such data indicate that OA is not only a very common condition but also that there is a significant unmet need for education for owners and pain management for dogs suffering from this condition.

The majority of dogs with osteoarthritis are managed using conservative and medical approaches. Since the 1990s, non-steroidal anti-inflammatory drugs (NSAIDs) have been approved for dogs to treat pain associated with osteoarthritis. Numerous studies document the effectiveness of NSAIDs in dogs (4–9), along with research on the adverse events (AEs) that may occur with their use (10, 11).

Meloxicam is a commonly used NSAID that was licensed in the 1990s for the treatment of canine osteoarthritis (OA) (9) and is used in many territories globally. Previous studies have reported the efficacy of meloxicam and also reported a gastrointestinal adverse event rate of 12% (12) and 15% (5).

In 2021, a novel class of analgesics was licensed in Europe and the United Kingdom for relieving pain associated with OA. Bedinvetmab (Librela^®^, Zoetis) is a fully canine monoclonal antibody that targets nerve growth factor (NGF) and has since been licensed in over 50 territories, including the United States, Canada, Latin America, Asia, and Australia. Nerve growth factor was identified as a key mediator of joint pain in preclinical animal models of OA (13) and is a neurotrophic factor involved in pain signal transduction and nociceptor receptor gene expression, playing an important role in pain signalling in OA. Nerve growth factor is a soluble protein released by mast cells (14), macrophages (15), and chondrocytes (14). It binds to the high-affinity NGF-specific tropomyosin receptor kinase A (TrkA) on peripheral nociceptors, potentially leading to both peripheral and central sensitisation.

Furthermore, when NGF binds to TrkA receptors on immune cells, it triggers the release of additional pro-inflammatory mediators, including NGF itself (16). In the periphery, NGF also binds to TrkA receptors on mast cells and other immune cells, resulting in the release of inflammatory mediators such as histamine, serotonin, and NGF itself. Thus, NGF can trigger peripheral sensitisation and sensitise adjacent nociceptive neurones through the release of these inflammatory mediators (17–20). The nerve growth factor also drives neoinnervation in the synovium and subchondral bone (14). Blocking NGF binding to TrkA has been demonstrated to reduce OA pain in preclinical models (13), and subsequent clinical trials have confirmed the efficacy of anti-NGF antibodies in humans (21), cats (22), and dogs (23–25) with OA. In addition, using a validated Health-Related Quality of Life instrument, a clinical study conducted in the United Kingdom reported that bedinvetmab improved the quality of life in dogs across multiple domains (26).

The European and American field trials of bedinvetmab compared dogs with osteoarthritis receiving bedinvetmab to those receiving a placebo (24, 25). Whilst these studies demonstrated efficacy for regulatory purposes, clinicians are also interested in the efficacy and safety of new medicines when compared to existing ones.

Three published pain guidelines [AAHA (27), WSAVA (28), COAST (29)] recommend both NSAIDs and anti-NGF monoclonal antibodies as appropriate first-line treatments for dogs with OA pain. Furthermore, bedinvetmab was recommended (by majority consensus) to manage OA pain in dogs with moderate OA for a minimum of 2 months (COAST).

Accordingly, the study reported herein was designed to compare the efficacy and safety of bedinvetmab with that of the NSAID meloxicam in a randomised, open-label, parallel-group clinical trial involving dogs with OA over a 56-day treatment period. The authors hypothesised that bedinvetmab would exhibit superior efficacy and safety compared to meloxicam.

## Materials and methods

### Study design

This randomised, open-label, multicentre clinical comparator study was conducted following an ethical review by the Zoetis VMRD ethical review assessment committee (Study 2INTORCADPAIN01).

Eligible dogs participating through veterinary clinics were randomly assigned to one of two treatment groups based on the order in which they presented to the clinic. The plan was to have at least 46 evaluable cases in each treatment group, and no single clinic was to enrol more than 40% of the total number of evaluable cases. Each participating site was to attempt to enrol at least two complete blocks (four animals).

Sample size estimates were determined based on the assumption that bedinvetmab (Librela^®^, Zoetis) would be superior to meloxicam in terms of efficacy. The primary outcome measure was the Canine Orthopaedic Index (COI), a validated client-reported outcome measure (CROM) (30–32). Sample size estimates using the COI required several assumptions: first, published work indicated a standard deviation for COI in a population of dogs with OA to be “10” for the aggregate COI score (32) and that treatment with the NSAID carprofen resulted in a mean decrease in COI score of “12.”

In the absence of published data, it appeared likely that meloxicam would have similar efficacy to carprofen in terms of impact on the COI score (8). Similarly, without published data suggesting otherwise, we set an arbitrary clinically meaningful difference between meloxicam and bedinvetmab at 6 points on the COI. Considering a 10% dropout rate and targeting 80% power indicated that we would need to recruit group sizes of 46.

### Study population

Client-owned dogs were recruited from eight primary care veterinary practices in the United Kingdom between March 2023 and March 2024. The recruited cases included dogs with incident OA or those with previously diagnosed OA who were receiving only nutraceuticals and were considered eligible for screening. Practice raised awareness of the study amongst their existing client base using provided resources, including in-clinic posters, websites, and social media posts. Clients could withdraw their dogs from the study for any reason at any time, and whenever possible, the reason for withdrawal was recorded.

### Inclusion criteria

Clinical and radiographic evidence of OA in dogs aged 1 year or older was confirmed in at least one major joint of either a thoracic (shoulder, elbow, carpus) or pelvic (hip, stifle, tarsus) limb during a screening visit. The dog was expected to benefit from continued treatment for a duration of 2 months for clinical signs of OA. The severity of at least one veterinary categorical assessment (general musculoskeletal condition, pain on palpation/manipulation of joint(s), lameness/weight bearing) was at least “moderate” (scale: clinically normal, mild, moderate, severe, nearly incapacitating) at both the screening and day 0. The COI, as assessed by the owner, was 26 or higher (maximum score 64). Veterinarians confirmed that the dogs were generally in good health, concomitant diseases were well controlled, and clinical pathology results (blood and urine) were satisfactory.

Specifically, the packed cell volume and total protein levels were within the normal range, with blood urea nitrogen (BUN) and creatinine also either within the normal range or a maximum of 10% above it. Alanine aminotransferase (ALT) and alkaline phosphatase (ALP) levels were either within the normal range or a maximum of three times the normal range. If ALT or ALP exceeded twice the upper limit of the normal range, bile acids were tested; if the bile acids were normal, the subject was included. If a dog was receiving any concomitant medications listed in Table 1, both the owner and the examining veterinarian had to agree to adhere to all withdrawal times, minimal usage, and frequency of usage. If a dog was receiving a conditionally allowed medication (Table 1), the dosage and dosage rate were not expected to change during the study. Dogs receiving nutraceuticals for OA [e.g., “joint” diets, glucosamine/chondroitin sulphate, green-lipped mussel extract, methylsulfonylmethane (MSM), fish oil, avocado soybean unsaponifiable (ASU)] were to continue at the same dosage throughout the study. The dog’s owner had to commit to evaluating their dog over a continuous period of 8 weeks. Additionally, the owner needed to anticipate a stable lifestyle (e.g., no impending major life events or periods of absence). The owner also had to agree to administer the medication according to instructions if randomised to the meloxicam group and to maintain the dog’s basic management (exercise, routines) throughout the 8-week period.

**Table 1:** Prohibited and conditionally allowed concomitant medications and withdrawal times.

Medication	Prohibited (P)/conditionally allowed (C)	Withdrawal time
Parenteral dexamethasone, betamethasone, methylprednisolone or triamcinolone	P	28 days
Corticosteroids of any type (oral, injectable or topical)	P	28 days
Antibiotic drugs with neuromuscular blocking properties	P	14 days
Other drugs considered analgesics	P	14 days
Other medicines with no known analgesic or anti-inflammatory conditions	C	n/a

n/a, not applicable.

### Exclusion criteria

The dogs excluded from the study included those enrolled in a clinical trial ≤30 days before day 0 or involving an injectable product <90 days before day 0; those intended for breeding, lactating, or pregnant; and those with lameness due to primary immunologic, neurologic, infectious, or neoplastic conditions. Additionally, dogs with ligament ruptures in the last 12 months or non-healed fractures were excluded. Dogs with a history of injury leading to neurologic deficits or intervertebral disc disease (if expected to interfere with efficacy assessment) were also excluded, as were those administered prohibited medication (see Table 1). The use of conditionally allowed medication was allowed under specific conditions regarding minimal use and frequency (see Table 1). However, dogs receiving any of the prohibited medications listed in Table 1 were excluded unless they had completed the respective withdrawal times. Further exclusions included dogs with conditions likely to require surgical intervention during the study; those whose lameness was known to be associated with neoplasia, primary neurologic or immunologic disorders (e.g., immune-mediated polyarthritis), infections (e.g., septic joint), recent joint trauma, or non-healed fractures. Dogs that had started a physical therapy programme less than 8 weeks before screening or a weight loss programme less than 8 weeks before screening (unless on a stable weight loss regimen) were also excluded. Finally, dogs with known hypersensitivity to the active substances or any excipients were ineligible for enrolment.

### Randomisation

Enrolled dogs were randomised in a 1:1 ratio to receive either bedinvetmab or meloxicam, using a randomised complete block design with a one-way treatment structure that was replicated across multiple clinics. All data collection and randomisation for the study were conducted using the Castor electronic data collection software platform (Castor EDC, Amsterdam, Netherlands).

Dogs were randomly assigned to groups based on their order of entry into the clinic study and the randomisation provided by the Castor platform. Within each site, blocks of two dogs were created according to the order of enrolment. In each block, dogs were randomly allocated to either meloxicam or bedinvetmab. Day 0 was the day a dog received its first dose.

### Treatment administration

For dogs randomised to bedinvetmab, the UK dosage chart (Supplementary material) ensured a bedinvetmab dosage of 0.5 to 1.0 mg/kg using a ready-to-use formulation in single-use 1 mL vials (vial strengths: 5, 10, 15, 20, or 30 mg/mL). The use of the dose chart results in a minimum target dose of bedinvetmab of 0.5 mg/kg (0.23 mg/lb). Both doses of bedinvetmab (day 1 and day 28) were administered by the attending veterinarian. Dogs randomised to meloxicam were administered an initial dose of 0.2 mg/kg by subcutaneous injection on day 1 from the veterinarian, and an oral meloxicam suspension was dispensed for administration at 0.1 mg/kg daily for the remainder of the study period (an additional 55 days) by the owner.

### Study schedule

A screening visit took place 0–21 days before day 1. The screening included the completion of the COI by the owner, a veterinary categorical assessment by the veterinarian, haematology, blood biochemistry, urinalysis, and a radiographic examination to confirm the presence of OA. Baseline COI data collection took place on day 1, prior to enrolment. Four additional clinic visits followed day 1, specifically on days 14, 28, 42, and 56. Dogs receiving bedinvetmab were dosed by the veterinarian on days 1 and 28. A veterinary physical examination was conducted at each visit, during which veterinarians completed the veterinary categorical assessment and documented any AEs or medications, if applicable. Within 3 days of each visit, owners, blinded to their prior assessments, completed the COI electronically via the Castor EDC platform. At the final clinic visit (day 56 ± 3 days), in accordance with recommended best practice for dogs treated with NSAIDs (33), particularly those on meloxicam, blood was collected for haematology and serum biochemistry, and urine was collected for urine specific gravity.

### Escape clause and the use of rescue and prohibited therapies

A dog could be withdrawn from the study by the veterinarian or owner at any time. Prohibited or conditionally allowed medications could be administered prior to withdrawal, if necessary, for animal welfare reasons. “Rescue” treatment for an OA-related issue (e.g., lack of efficacy) was considered a prohibited treatment. A “prohibited” treatment that was not classified as rescue treatment was one that could interfere with efficacy assessments and was used for reasons unrelated to OA.

### Efficacy outcome measures

The primary measure of efficacy was the change from the baseline COI score. Dogs with protocol deviations affecting integrity or data collection were excluded from the efficacy analyses. After enrolment, dogs were withdrawn from the study for using prohibited treatments. Dogs withdrawn for loss of efficacy or rescue treatment were included in the intent-to-treat analyses.

### Safety measures

For safety, AEs were recorded and categorised according to the Veterinary Dictionary for Drug Regulatory Activities (VeDDRA) (34). For dogs that received meloxicam, clinical pathology data on day 56 were compared to those collected on day 1.

### Compliance

Compliance with the administration of bedinvetmab was ensured by accurately recording a successful subcutaneous injection of the correct vial size on the case report form (CRF) within the Castor EDC platform. Similarly, compliance with administering the loading dose of meloxicam and dispensing the correct dose and bottle size of meloxicam was recorded in a similar manner. Additionally, the owner’s compliance in dosing meloxicam at home was evaluated by weighing the bottle of meloxicam during each visit.

### Statistical analysis

Data analysis was conducted using R (version 4.2, R Foundation for Statistical Computing) and MLwiN (version 3.10, Centre for Multilevel Modelling, University of Bristol) on a “per protocol” and “intent to treat” basis. For the intent-to-treat analysis, missing data were imputed using multiple imputations by chained equations with the R package *mice.* The COI data were analysed separately using a general linear mixed effects model for repeated measures, with the change in COI from baseline as the outcome. The initial COI score served as a covariate, incorporating random effects of site and dog (for repeated measures) and fixed effects of treatment and visit.

## Results

### Demographic data

**Figure 1:**
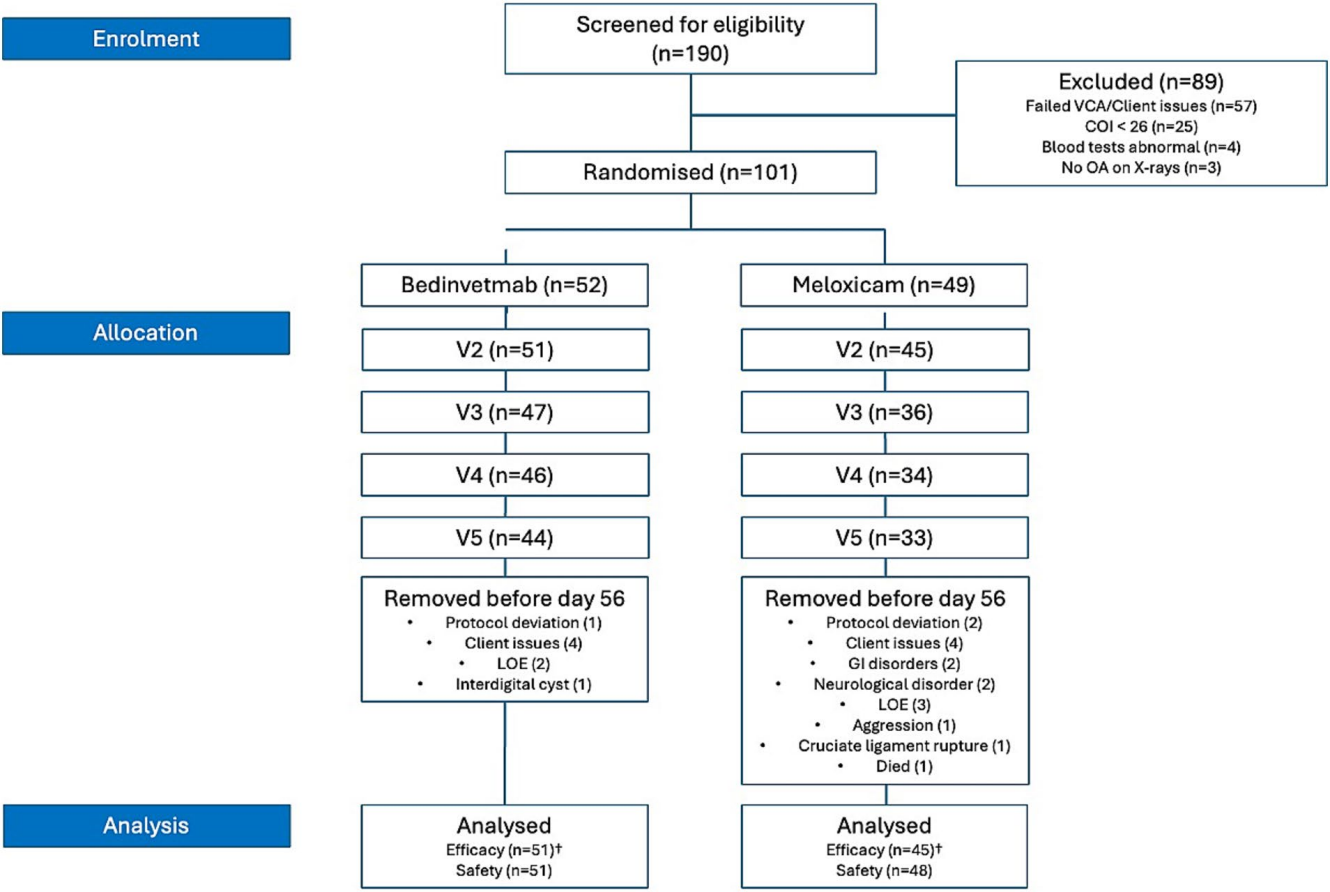
CONSORT flow diagram depicting all cases recruited for the 2-month study. *n*, number; COI, Canine Orthopaedic Index; VCA, Veterinary Categorical Assessment; LOE, lack of efficacy. †Data excluded due to protocol deviations (i.e., eligibility criteria not met, owner did not complete the COI assessment, animal withdrawn for developing an unrelated medical or surgical condition, owner non-compliance/lost to follow-up, or administration of prohibited treatments) clarifies the cases excluded from the efficacy analysis. All animals were included in the safety data analysis.

Eligibility criteria were not met for 89 of the 190 dogs screened; consequently, 101 dogs (meloxicam, *n* = 49; bedinvetmab, *n* = 52) were randomised (Tables 2, 3; Figure 1). A total of 25 dogs were excluded from this study because their COI scores were below 26 at the screening visit. Two dogs were mistakenly included in randomisation as their initial visit COI was less than 26; these dogs were not considered in any further analyses (including the intent-to-treat analysis), resulting in a total of 99 dogs available for analysis. The Labrador Retriever was the predominant breed (*n* = 27; 27%), followed by the Cocker Spaniel (*n* = 6; 6%) and the Border Collie (*n* = 4; 4%). No other individual pure breeds comprised ≥3% of the total, and 21 dogs (21%) were crossbreeds. The most common index joints in the study population were the hip (*n* = 41; bedinvetmab, *n* = 22; meloxicam, *n* = 19), followed by the elbow (*n* = 33; bedinvetmab, *n* = 17; meloxicam, *n* = 16). The remainder (*n* = 25) had OA of the stifle, shoulder, or tarsus.

**Table 2:** Summary of treatment groups and dosing regimens.

Treatment group	Number of dogs	Test article	Dose (mg/kg)	Route of administration	Day of treatment^*^	Days of study visits^*^
T01	52	Librela	0.5–1**	SC	Day 0, 28	Days 0, 14, 28, 42, 56
T02	49	Meloxicam oral suspension	0.2 on Day 1 and 0.1 thereafter***	Oral	Daily Days 0–56	Days 0, 14, 28, 42, 56

*±3 days (Days 14, 28), ±3 days (Days 42, 56) (and activities associated with visits).

**The actual dose of Librela varied between 0.5 and 1.0 mg/kg depending upon the individual dog’s body weight according to the Dosing Table.

***Initial treatment is a single dose of 0.2 mg meloxicam/kg body weight on the first day. Treatment was continued once daily by oral administration (at 24-h intervals) at a maintenance dose of 0.1 mg meloxicam/kg body weight.

**Table 3:** Demographics of enrolled dogs at day 0.

	Meloxicam (*n* = 49)	Bedinvetmab (*n* = 52)	Total (*n* = 101)
Breed distribution			
Crossbreed	9 (18.4)	12 (23.0)	21
Labrador	15 (30.6)	12 (23.0)	27
Other	25 (51.0)	28 (54.0)	53
Sex distribution			
Male	10	5	15
Male neutered	13	23	36
Female	0	3	3
Female neutered	26	21	47
Age (years)	10.6 ± 2.6 (4–16)	10.3 ± 2.6 (1–15)	10.5 ± 2.6 (1–16)
Bodyweight (kg)	25.9 ± 9.8 (5.6–47.0)	22.0 ± 9.0 (9.7–53.0)	23.9 ± 9.5 (5.6–53.0)
Body condition score	5.6 ± 0.92 (4–8)	5.7 ± 1.2 (2–9)	5.6 ± 1.1 (2–9)
Pre-treatment COI	39.7 ± 9.2 (21–56)	40.2 ± 9.7 (26–59)	40.0 ± 9.4 (26–59)

Data are presented as *n* (%) or mean ± standard deviation (range). *n* represents the number of animals, including all enrolled animals, whilst excluding those with protocol deviations from the analysis.

### Efficacy assessment

Of the enrolled dogs, 23 were withdrawn prior to visit 5 (day 56) (meloxicam, *n* = 15; bedinvetmab, *n* = 8) (Table 4). Five dogs were withdrawn due to loss of efficacy (meloxicam, *n* = 3; bedinvetmab, *n* = 2). Six animals were withdrawn for medical or surgical conditions deemed unrelated to the test article (meloxicam, *n* = 5; bedinvetmab, *n* = 1), and six were withdrawn due to owner non-compliance or because they were lost to follow-up. Data were excluded for protocol deviations related to visits outside the permitted window (meloxicam, *n* = 1). No dogs were withdrawn due to the administration of prohibited medication.

**Table 4:** Overview of dogs with osteoarthritis that were administered either daily oral meloxicam or monthly subcutaneous injections of bedinvetmab for 2 months and were withdrawn from the study before the day 56 visit.

Visit	Group	
Visit 1 (Day 0)	Meloxicam (*n* = 49)	Bedinvetmab (*n* = 52)
	**Cumulative total number of dogs withdrawn before day 14 visit = 5**	**Cumulative total number of dogs withdrawn before day 14 visit = 1**
	1 case was withdrawn because it was incorrectly randomised, two clients failed to arrive, one for major protocol deviation (visit date), and one dog died (suspected malignancy)	One case was withdrawn because it was incorrectly randomised
Visit 2 (Day 14)	Meloxicam (*n* = 44)	Bedinvetmab (*n* = 51)
	**Cumulative total number of dogs withdrawn before day 28 visit = 13**	**Cumulative total number of dogs withdrawn before day 28 visit = 5**
	One client failed to arrive; two cases were withdrawn because of developing gastrointestinal disorders, two for LOE, two for neurological deficits in the pelvic limbs, and one for aggression	Three cases were withdrawn because of client issues and one because of LOE
Visit 3 (Day 28)	Meloxicam (*n* = 36)	Bedinvetmab (*n* = 47)
	**Cumulative total number of dogs withdrawn before day 42 visit = 15**	**Cumulative total number of dogs withdrawn before day 42 visit = 6**
	One case was withdrawn because of acute onset lameness associated with cruciate ligament rupture, and one because the client failed to arrive	One case was withdrawn because of LOE
Visit 4 (Day 42)	Meloxicam (*n* = 34)	Bedinvetmab (*n* = 46)
	**Cumulative total number of dogs withdrawn before day 56 visit = 16**	**Cumulative total number of dogs withdrawn before day 56 visit = 8**
	One case was withdrawn because of LOE	One client failed to arrive, and one case was withdrawn because of an interdigital cyst
Visit 5 (Day 56)	Meloxicam (*n* = 33)	Bedinvetmab (*n* = 44)

Data from cases with protocol deviations were excluded from the efficacy analysis, whilst all animals were included in the safety analysis.

### Owner assessments (COI)

#### Per protocol analysis

**Figure 2:**
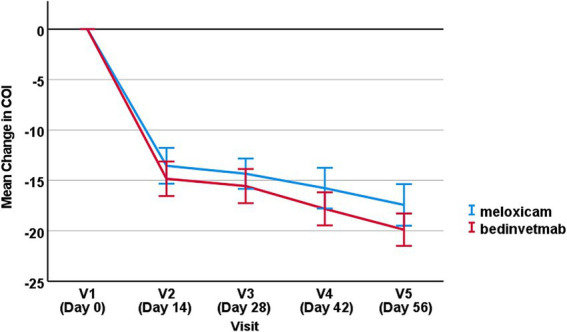
Mean change in COI score from baseline (±SEM) for visits 2–5 for dogs treated with bedinvetmab and those treated with meloxicam.

For the change in COI from baseline, there was no significant effect of treatment (i.e., meloxicam *vs.* bedinvetmab), with *p* = 0.57 (Figure 2). There was a significant effect of visit, with all later visits showing a significantly greater reduction in COI compared to Day 14 (*p* < 0.001), indicating that clinical status in both groups improved significantly, as reflected by owner COI scores.

#### Intent-to-treat analysis

For the change in COI from baseline, there was no significant effect of treatment (i.e., meloxicam *vs.* bedinvetmab) with *p* = 0.42. However, a significant effect of visit was observed, with Days 42 and 56 showing a significantly greater reduction in COI compared to Day 14 (*p* < 0.001).

### Safety assessment

#### AEs and concomitant medications

Three AEs were associated with clients (two incidents related to clients’ health and one involving a client who lost their means of transport); these AEs were excluded from further analysis.

#### Per protocol analysis

At least one AE was reported for 17 dogs in the meloxicam group and four dogs in the bedinvetmab group (see Tables 5, 6). The most commonly reported AEs were gastrointestinal disorders, including diarrhoea, and emesis, which occurred in the meloxicam group. Based on these AE rates, the number needed to harm for meloxicam compared to bedinvetmab was 5 (95% Confidence Interval 2.8–27.2). Two dogs in the meloxicam group were euthanised: one between visits 1 and 2 due to suspected malignancy (not confirmed) and the other between visits 2 and 3 due to aggression that resulted in a bite injury to a person.

**Table 5:** AEs in dogs with osteoarthritis receiving daily doses of meloxicam or monthly subcutaneous injections of bedinvetmab (the bedinvetmab group) occurring at least once.

System organ class clinical sign (VeDDRA)	Group	
	Meloxicam (*n* = 49)	Bedinvetmab (*n* = 52)
Any AE	17	4
Skin and appendage disorders (e.g., pruritus, dermatitis)	1	1
Digestive tract disorders (e.g., diarrhoea, emesis)	8	0
Systemic disorders (e.g., polydipsia, lethargy)	1	1
Musculoskeletal disorders (e.g., lameness, joint pain)	3	2
Behavioural disorders (e.g., aggression)	1	0
Neurological disorders (e.g., ataxia)	3	0

AEs are categorised by the frequency of clinical signs, according to the Veterinary Dictionary for Drug Regulatory Activities (VeDDRA) system organ class classification on a per-animal basis^∗^. Data are presented as n (%); *n* represents the number of dogs (all animals enrolled).

*Occurrence is calculated on a per-animal basis; regardless of how many observations of the same AE are recorded for a dog, only a single observation contributes to the occurrence calculation.

**Table 6:** AEs occurring at least once in dogs with osteoarthritis receiving daily doses of meloxicam or monthly subcutaneous injections of bedinvetmab.

Preferred term clinical sign (VeDDRA)	Group	
	Meloxicam (*n* = 49)	Bedinvetmab (*n* = 52)
Any AE	17 (34.7)	4 (7.7)
Diarrhoea	5 (10.2)	0 (0.0)
Lameness	3 (6.1)	2 (3.8)
Emesis	3 (6.1)	0 (0.0)
Pruritus	1 (2.0)	0 (0.0)
Dermal cyst	0 (0.0)	1 (1.9)
Anorexia	1 (2.0)	0 (0.0)
Polydipsia	0 (0.0)	1 (1.9)
Ataxia	2 (4.0)	0 (0.0)
Proprioception abnormality	1 (2.0)	0 (0.0)
Aggression	1 (2.0)	0 (0.0)

AEs are listed by the frequency of clinical signs based on the Veterinary Dictionary for Drug Regulatory Activities (VeDDRA) preferred term classification on a per-animal basis^∗^. Data are presented as n (%); n represents the number of dogs (all animals enrolled).

*Occurrence is calculated on a per-animal basis; regardless of how many observations of the same AE are recorded for a dog, only a single observation contributes to the occurrence calculation.

### Clinical pathology

At screening, the mean and median values for all haematology and serum chemistry analytes in both groups fell within the reference ranges for the measured analytes in 98 of the 101 enrolled dogs. In three dogs, ALT and/or ALP levels were elevated during the screening visit, but bile acids remained within normal limits, and the dogs were enrolled according to the protocol.

Between days 1 and 56, changes in clinical pathology results (either increases or decreases compared to pre-treatment) were observed in the meloxicam group; however, only the changes in creatinine were statistically significant. Creatinine concentrations increased significantly from day 1 to day 56 (*p* = 0.031). No significant changes were noted in BUN (*p* = 0.32) or urine-specific gravity (*p* = 0.27). Liver enzymes, including ALT and ALP, did not exhibit significant changes from day 1 to day 56 (*p* = 0.34 and *p* = 0.10, respectively). No other significant changes in the measured parameters were observed.

### Compliance

Compliance for bedinvetmab was recorded at 100% because all investigators administered the subcutaneous injection in the clinic and documented it in the EDC system. For meloxicam, analysing the data proved challenging. We requested that the meloxicam bottle be weighed at each visit, as this would allow us to estimate compliance by recording the weight loss between visits and the product’s specific gravity. Data indicated that five different brands of meloxicam were used in the study: Metacam (Boehringer Ingelheim), Loxicom (Norbrook Laboratories Ltd.), Rheumocam (Chanelle Pharma), Meloxidyl (Ceva Animal Health Ltd.), and Inflacam (Virbac Ltd.). Whilst the specific gravity for Metacam is published (1.56), specific gravity values for other brands were not always available, and we were advised that these values could vary from batch to batch. The best analysis we could perform suggested an average underdosing of meloxicam by 32%, but the confidence in this result cannot be considered high.

## Discussion

The canine population in this study was similar to that in other studies examining the efficacy of meloxicam (9, 35) and bedinvetmab (24, 25) in dogs with OA pain. Dogs identified with incident OA in primary care settings tend to be older, as demonstrated in this study, with a mean age exceeding 10 years. We excluded dogs younger than 1 year because bedinvetmab is not approved for this age group. The most prevalent breed in this study was the Labrador Retriever, a popular breed known for its predisposition to OA in the elbow and hip joints (36–38). The most common index joints in the studied dogs were the hip joint, followed by the elbow joint, showing a similar distribution between both groups.

Although no published data exist on the relationship between COI scores and the severity of OA as assessed by other measures, the dogs in this study were required to have a minimum baseline score of 26 or higher. Additionally, one category in the Veterinary Categorical Assessment needed to be rated as at least moderate, and based on that assessment, the dogs in this study should be considered to have moderate or more severe osteoarthritis.

Outcome measures for canine OA have evolved over time, with validated CROMs being used more frequently. In recent years, regulatory studies in canine OA have used the Canine Brief Pain Inventory (CBPI) (24, 25, 39) as a primary outcome measure because this instrument has established thresholds for defining responders and non-responders (40). However, a recent COSMIN-based review concluded that two other CROMs, namely the COI and the Liverpool Osteoarthritis in Dogs (LOAD), also provided sufficient evidence for their use in evaluating dogs with osteoarthritis (41).

“Minimal clinically important differences” (MCIDs) have recently been published for COI and LOAD (42, 43). In veterinary medicine, the MCID represents the smallest improvement considered valuable by a client. The availability of MCID estimates has facilitated the use of these instruments in clinical studies, such as the one reported herein, and should now enable their use in regulatory studies. Since the CBPI is designed to provide *mean* scores for multiple items in the “pain severity score” and “pain interference score” categories, it may be less responsive to changes in clinical status compared to tools like COI and LOAD, which are aggregate scores of several items. Some evidence in the published literature supports this idea (23).

The MCID for COI has been estimated at 14 (42, 43). Because we wanted to detect a reduction in COI scores equal to or greater than the MCID and to assess whether one treatment was superior to the other, we established an inclusion criterion of a minimum COI score of 26 at day 0. Data on the use of COI in clinical research remain relatively limited (43–53), as this instrument has only been validated in recent years (30–32), whereas the CBPI and LOAD scales have been validated for considerably longer periods (54–57). Nevertheless, the results of our study suggest that this threshold of 26 allowed us to detect a significant reduction in COI scores. However, it has also led to attrition during the screening process, with 25 dogs being excluded due to COI scores below 26.

In the absence of published information, for sample size estimates in this study, we established a threshold of a 6-point difference in COI scores to indicate a clinically meaningful difference between bedinvetmab and meloxicam. A *post-hoc* analysis of another dataset available to us (43) supported this decision. In that previous study, changes in COI scores were categorised based on client responses to an anchor question, with the average difference between the “somewhat better” and “much better” groups being 4.5 points on the COI (rounded up 5 points) (43).

The primary outcome measure in this study was defined as the “change in COI score from baseline.” Both treatments resulted in a statistically significant reduction in COI scores over time, and bedinvetmab performed equally well to meloxicam, with a non-significant difference in efficacy between treatments.

Although the difference was non-significant at all timepoints, the mean reduction in COI scores was greater in the bedinvetmab group than in the meloxicam group at each sampling point.

On day 14 (visit 2), on average, the dogs in the bedinvetmab group experienced a reduction in COI scores of 14 points, which is equal to the MCID. In contrast, the dogs in the meloxicam group did not see a decrease in COI scores that equalled or exceeded the MCID (the mean change in COI score for meloxicam was 12.9 points). Throughout the study, both groups experienced further reductions in mean change from baseline COI scores, with the total mean reduction for bedinvetmab being 19.7 points and for meloxicam being 17.1 points. These data support the use of analgesics for managing OA pain for a duration sufficient to observe maximum benefits for affected dogs. The phenomenon of increasing benefits from continuous analgesia has been previously reported (4, 5), and expert guidelines also recommend treatment for a minimum of 1 to 2 months (29).

There were more AEs in the meloxicam group than in the bedinvetmab group. The most common category of AE in the meloxicam group was digestive tract disorders (diarrhoea and emesis), and it is well recognised that NSAIDs, such as meloxicam, can be associated with such AEs (10, 11, 58, 59). None of these gastrointestinal AEs were categorised as “severe,” but three were significant enough that a decision was made to withdraw the subject from the study. The rate of gastrointestinal AEs observed in this study was consistent with previous studies, which reported 12–15% of gastrointestinal AEs associated with meloxicam treatment (5, 12).

We also found a statistically significant rise in blood creatinine concentrations in the meloxicam group. NSAIDs, including meloxicam, are known to affect renal function; however, to our knowledge, a rise in creatinine associated with daily meloxicam treatment has not been reported previously. Another study examined blood biochemistry in dogs with hip osteoarthritis before and after 30 days of meloxicam treatment but did not identify any significant changes (60). None of the dogs in this study had blood creatinine levels at day 56 that exceeded the normal reference range, and none developed clinical signs of renal disease. Additionally, other related parameters, such as BUN and urine-specific gravity, did not exhibit significant changes during the study.

Assessing compliance for meloxicam in this study is challenging due to likely variations in specific gravity values amongst different generic brands. An examination of the excipients in these products revealed considerable inconsistencies between brands, which contribute to the variation in specific gravity. Nevertheless, there remains a possibility of some loss of compliance with daily oral medication administered by clients at home. Underdosing may lead to a reduction in efficacy, but conversely, it might also reduce AEs. Although this was a clinical trial with visits every 14 days, leading one to expect clients to be more vigilant regarding compliance, previous research indicates that median compliance amongst dog owners for chronic conditions can be as low as 56% (61). For chronic conditions such as OA, compliance is likely a crucial factor in providing effective pain relief and improving animal welfare.

There were only four AEs in the bedinvetmab group. Two of these were cases of lack of efficacy (LOE), compared to three such AEs in the meloxicam. One AE involved an interdigital cyst, which was likely unrelated to the test compound but interfered with mobility and the owner’s ability to use the COI effectively. As a result, the dog was withdrawn from the study. The remaining AE was polydipsia. Following the regulatory approval of bedinvetmab, pharmacovigilance data have revealed polydipsia (and associated polyuria) as a rare AE associated with bedinvetmab administration,^1^ which is now included in the summary of product characteristics in the United Kingdom and European Union. A rare AE is defined as occurring “1–10 animals per 10,000 animals treated.” At the time of writing, the authors understand that the pathophysiology of polydipsia associated with the administration of bedinvetmab remains unknown. In this study, the client considered the issue mild and tolerable and chose not to withdraw the dog from the study due to the observed efficacy of the product. Polydipsia and polyuria were not reported as statistically significant AEs in clinical trials of tanezumab, a similar medicine for humans (62), highlighting the species-specific nature of AEs.

A phenomenon of the current era is the emergence of social media groups linked to the launch of new medicines. Such groups can grow large but often lack rigorous analysis, context regarding the number of treated dogs, verified veterinarian oversight for pets’ overall medical conditions, and considerations for age-matched control populations. The quality of data is higher in the post-registration pharmacovigilance structures and methods of regulatory authorities such as the Food and Drug Administration (FDA) in the United States, the European Medicines Agency (EMA) in the European Union, and the Veterinary Medicines Directorate (VMD) in the United Kingdom. Nevertheless, these social media groups can have an impact (63). Anecdotally, they have reported neurological signs associated with bedinvetmab administration. No such AEs were noted in the study reported herein, but it is interesting to note that three dogs in the meloxicam group experienced neurological AEs during the study, including two with ataxia and loss of proprioception.

All dogs in the study were clinically screened for neurological disorders at the beginning, and such comorbidities were grounds for exclusion. Acute onset ataxia is recognised as a common issue in older dogs (64), as is vestibular syndrome (65). In an age-associated disease such as OA, it is important to consider suspected adverse treatment events in the context of the background event rate within the target population. It should be noted that in the overall dog population, the proportion of dogs with a history of neurologic conditions increases from less than 1% in puppies and adolescents to 12% in senior dogs, with larger breeds showing a steeper increase across age compared to dogs under 40 kg (66). The dogs in this study had an average age of over 10 years, and the older age range of dogs in studies such as this probably makes such AEs more likely; further studies are required to define the expected AE rates for various body systems in older dog populations.

Overall, from a safety perspective, this study demonstrated a lower AE rate in dogs treated with bedinvetmab (Librela, Zoetis) compared to those treated with meloxicam during a 56-day treatment period. Furthermore, the number needed to harm for meloxicam, in relation to bedinvetmab, was five. In other words, on average, five patients would need to be treated with meloxicam for one to experience harm beyond what occurs with bedinvetmab. However, it must be acknowledged that this study lasted for only 56 days, which limits the safety data conclusions. Longer-term treatment with NSAIDs compared to bedinvetmab will need further research to document any differences during extended treatment periods.

In summary, this is the first comparator clinical trial of bedinvetmab versus the NSAID meloxicam for managing pain associated with canine osteoarthritis. The results indicate that bedinvetmab performed comparably to meloxicam in effectively managing OA pain, starting on day 14. Both products demonstrated increasing efficacy over the 56-day period. Although a positive trend was observed for bedinvetmab (the bedinvetmab group exhibited a larger mean reduction in COI scores), there was no significant difference in efficacy between the two products. Both resulted in a statistically significant reduction in COI scores from baseline. This study demonstrated that bedinvetmab had a lower AE rate compared to meloxicam. These data will be valuable for clinicians and owners when making decisions regarding analgesic medications for managing canine osteoarthritis.

## Data Availability

The datasets presented in this article are not readily available because data will be held by Zoetis. Requests to access the datasets should be directed to Oliver Knesl, Oliver.knesl@zoetis.com.

## Footnotes

^1^ https://www.vmd.defra.gov.uk/productinformationdatabase/product/A010534
