# Supplementary material for: A randomised, parallel-group clinical trial comparing bedinvetmab to meloxicam for the management of canine osteoarthritis
Source: Front Vet Sci. 2025 Mar 24;12:1502218. doi: 10.3389/fvets.2025.1502218 (PMC11974340; doi:10.3389/fvets.2025.1502218)
Supplement: Supplementary file 1 [file Table_1.DOCX]

**Supplementary material**

Dosing chart for bedinvetmab (Librela, Zoetis) supplied to all investigators on the Castor EDC platform


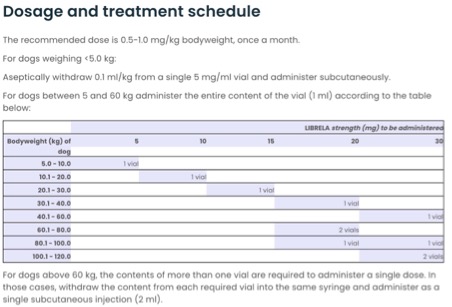


* Chart used with permission from Zoetis
